# Retraction of: Silencing of circular RNA_0000326 inhibits cervical cancer cell proliferation, migration and invasion by boosting microRNA-338-3p-dependent down-regulation of CDK4

**DOI:** 10.18632/aging.206014

**Published:** 2024-06-30

**Authors:** Zhaoxin Wang, Chenchen Ren, Li Yang, Xiaoan Zhang, Jiaxi Liu, Yuanhang Zhu, Dongyuan Jiang

**Affiliations:** 1 Department of Obstetrics and Gynecology, The Third Affiliated Hospital of Zhengzhou University, Zhengzhou 450052, P. R. China; 2 Academy of Medical Science, Zhengzhou University, Zhengzhou 450052, P. R. China; 3 Department of Imaging, The Third Affiliated Hospital of Zhengzhou University, Zhengzhou 450052, P. R. China

**Keywords:** cervical cancer, circular RNA, circ_0000326, microRNA-338-3p, cyclin-dependent kinase 4

**This article has been retracted:** Aging has completed its investigation of this paper. We found internal and external duplications and manipulations, which include rescaling and cropping. We found that there was overlap between the transwell experiments shown in **Figure 3A** depicting the invasiveness of HeLa cells transfected with sh-circ-0000367 and **Figure 7A** depicting the motility of HeLa cells transfected with mir-338-3p-mimic. In addition, the immunofluorescent image of HeLa cells transfected with mir-338-3p-mimic in the EDU proliferation assay presented in **Figure 6B** was previously published in an unrelated paper [1]. Erroneously, the ruler in **Figure 8B** is identical to the ruler used in earlier published papers [2, 3] and appeared later in tumor images in unrelated papers [4, 5]. All these inconsistencies have led to a loss of confidence in the integrity of the results presented in this article. In light of the above issues, the Aging Editors retract this article. First author does not agree to this retraction. None of the other authors have responded to any correspondence from the Scientific Integrity Office about this retraction. Aging notified authors’ Institutions about this retraction and added authors names to the Editorial Warning list.
